# Intercalation-Induced Amorphization Boosts Aqueous Magnesium-Ion Storage

**DOI:** 10.1021/acs.chemmater.5c03477

**Published:** 2026-04-21

**Authors:** Tongxin Zhou, Divakar Arumugam, A. M. Milinda Abeykoon, Gihan Kwon, Cheng-Hung Lin, Lihua Zhang, Xiaowei Teng

**Affiliations:** † Department of Chemical Engineering, 8718 Worcester Polytechnic Institute, 100 Institute Road, Worcester, Massachusetts 01609, United States; ‡ National Synchrotron Light Source II, 8099 Brookhaven National Laboratory, Upton, New York 11973, United States; § Center for Functional Nanomaterials, Brookhaven National Laboratory, Upton, New York 11973, United States

## Abstract

The design of aqueous battery cathode materials that can store divalent ions with high capacity and satisfactory reversibility is of great technical importance and challenge. Here, we report that divalent Mg^2+^ storage is facilitated by an intercalation-induced amorphization of vanadate electrode materials. Electrokinetic analyses and in situ synchrotron X-ray diffraction and absorption spectroscopy collectively demonstrate that vanadate layered materials (Li–V_3_O_8_) undergo a structural transformation to amorphization induced by Mg^2+^ intercalation, and a reversible restoration of crystalline structure upon Mg^2+^ deintercalation. Debye scattering simulations suggest that intercalation-induced turbostratic disorder, especially random rotations, translational shifts, oscillatory motions, or varied interlayer spacing of adjacent V–O molecular layers, could be responsible for the observed amorphization. The highly distorted local structure, in turn, facilitates Mg^2+^ intercalation across the vanadate electrode materials, responsible for nearly 3/7 of the total Mg^2+^ ions intercalated. The study presented reveals an intriguing relationship between ion transport and the reversible amorphization-to-crystallization dynamics it induces, opening a paradigm for designing advanced aqueous battery electrodes.

## Graphical abstract

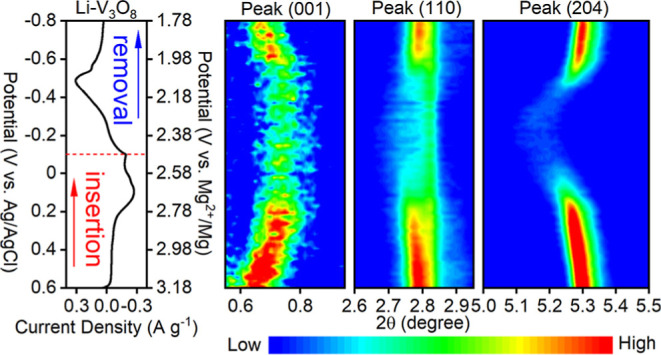

## Introduction

The rapid expansion of renewable energy generation has created an urgent demand for long-duration energy storage (LDES) technologies capable of storing electricity for days or beyond. Such systems are essential for stabilizing the grid during extreme weather events. Existing lithium-ion battery (LIB) technologies, while mature, remain cost-prohibitive for large-scale deployment, offer limited discharge durations of only a few hours, and pose fire hazards due to their flammable electrolytes. Safe, cost-effective, and sustainable LDES technologies are therefore crucial to achieving grid resiliency and deep decarbonization. Aqueous rechargeable batteries have garnered widespread attention for grid-scale stationary applications due to their distinct advantages over nonaqueous counterparts, including the abundance of raw materials, safety, environmental friendliness, high ionic conductivity, and the avoidance of a stringent air- and moisture-free cell manufacturing environment.^1,2^ Aqueous batteries are becoming crucial for integrating renewable energy sources and developing sustainable power systems.

Magnesium (Mg) cations, characterized by their abundance, stability, nontoxicity, divalent valence, and small ionic radius, have attracted scientific interest as a promising charge carrier for rechargeable aqueous batteries.^3,4^ The divalent Mg^2+^ ion has a higher charge density (defined as the charge per unit volume) than monovalent ions (e.g., Li^+^, Na^+^) and other divalent counterparts such as Ca^2+^ and Zn^2+^ due to its smaller ionic radius.^5,6^ Therefore, the strong electrostatic interactions between Mg^2+^ and the host electrode material induce significant stress on the electrode lattice, leading to substantial volumetric changes during intercalation and deintercalation processes.^7^ Moreover, the slow diffusion of divalent ions within electrode materials hinders ion transport within the host electrode, thereby increasing the overpotential and ultimately impairing storage capacity.^6,8^ To overcome these issues, the decrease in crystallization that enables the production of disordered or even amorphous materials presents, in principle, a good opportunity to enhance multivalence ion storage. The random long-range networks in the highly disordered or amorphous phase provide ample isotropic ion diffusion routes and large numbers of point defects or planar defects that enhance the ion-intercalation capacity of the material.^9–11^ Furthermore, the highly porous structure of disordered or amorphous materials can often accommodate minor lattice distortions and volume changes during cation insertion/extraction, thereby enhancing cycling stability.^12^ For example, amorphization of molybdenum oxide has been recently reported using an electrochemical anodic oxidation strategy,^13^ where a disordered atomic arrangement allows multivalent molybdenum redox centers for simultaneous hosting multivalent cations in aqueous solutions, including H^+^, Li^+^, Mg^2+^, Ca^2+^, and Al^3+^, where a highly amorphous and porous electrode structure facilitates the fast ion transport via a pseudocapacitive storage mechanism.

For the layered electrode materials (e.g., transition-metal oxides, hydroxides, or dichalcogenides), introduction of turbostratic disorder, referring to the loss of long-range registry between successive layers (while the local in-plane order of each layer is still preserved), might be the most adopted design principle to improve the ionic transport and thus storage capacity.^14^ The turbostratic disorder primarily comprises adjacent layers rotating (rotational disorder), successive layers sliding (translational disorder), and/or randomly varying interlayer spacing (stacking faults). Turbostratic disorder often enhances ion intercalation by relaxing the rigid structural registry of layered electrodes, thereby reducing the high-energy barriers to ion transport.^15,16^ Moreover, unlike rigid stacking in ordered layered materials, disordered electrodes allow more flexible stacking, reducing stress accumulation during repeated intercalation and deintercalation and thereby improving cycling stability.^17^

On the other hand, when highly disordered local structures, including those induced by amorphization engineering, are employed for multivalent ion storage, the electrode materials undergo substantial structural deformation relative to their crystalline counterparts, which can lead to material degradation or leaching, particularly in vanadium-based materials.^12,18^ This drawback is one of the primary factors hindering the widespread adoption of highly disordered electrode materials for electrochemical systems that provide long-duration storage.^19^ Therefore, it is of utmost importance to break the trade-off between augmented disordered local structure for multivalent ion storage and alleviated electrode deformation for long-term stability. Addressing this challenge will significantly advance the development of aqueous energy storage.

To date, many efforts have been made to explore cathode materials for aqueous Mg^2+^ storage, including Prussian blue nickel hexacyanoferrate,^20^ LiV_2_(PO_4_)_3_,^21^ and Mn-based birnessite and spinel oxides,^22,23^ as well as polymorphs of V_2_O_5_.^24–26^ In particular, polymorphs of V_2_O_5_ layered materials have been intensively studied as cathode materials for aqueous metal ion batteries, for their wide valence range and tunable interlayer distance ranging from ∼4 Å to ∼22 Å by altering V–O polyhedra structures and intercalated species (e.g., cations, water, and organic molecules).^27,28^ This structural flexibility improves ion storage capacity by providing not only spacious interlayer spacing for ion transport but also abundant structural water molecules that help shield the charge of multivalent cations during electrochemical processes. Meanwhile, preintercalated ions such as Ca^2+^, Mg^2+^, and Mn^4+^ are believed to form “pillars” in between V–O molecular frameworks and improve the structural stability and transport kinetics of Mg^2+^ of V_2_O_5_ polymorphs.^25,26,29^ Despite reported performance improvements, these materials still do not resolve the trade-off between augmented electrode disorder for Mg^2+^ storage and compromised structural stability, which has been a lingering concern for multivalent ion storage in layered electrode materials.

Herein, we report the use of Li^+^ preintercalated vanadate (Li–V_3_O_8_) with an interlayer spacing of 11.3 Å to address the trade-off between disorder-driven high storage capacity and poor structural stability. The Li–V_3_O_8_ electrode exhibits excellent electrochemical performance for aqueous Mg^2+^ storage, with a discharge capacity of 140 mAh/g and a capacity retention of 75.2% after 100 cycles in a 0.1 M MgSO_4_ electrolyte. In situ X-ray diffraction (XRD) analysis and Debye scattering simulation are employed to elucidate the material’s structure and reaction mechanism, providing a basis for understanding its reported performance. We discovered an intriguing amorphization-crystallization transition in Li–V_3_O_8_ cathode materials, which is reversibly induced by the intercalation and deintercalation of Mg^2+^. More importantly, Mg^2+^ intercalation induces structural amorphization by introducing turbostratic disorder in the vanadate electrode, facilitating Mg^2+^ transport and improving storage capacity.

## Experimental Methods

### Material Synthesis

Li–V_3_O_8_ electrode materials are synthesized using commercially available α-V_2_O_5_ bulk materials as precursors. Specifically, α-V_2_O_5_ bulk powder (100 mg, Sigma-Aldrich, Inc.) was dispersed in 9 mL of Li_2_SO_4_ (0.5 M, Alfa Aesar) and stirred for 6 days. Subsequently, the resulting final product, Li–V_3_O_8_, was washed sequentially with 50 mL of deionized water and 50 mL of ethanol, then dried under vacuum overnight at room temperature.

### Electrochemical Measurements

Electrochemical measurements were conducted in homemade three-electrode half-cells using an electrochemical potentiostat (CH Instruments 660D/E). Cellulose-based filter paper (Whatman) was used as the separator, Pt wire was used as the counter electrode, and an Ag/AgCl reference electrode filled with 3 M KCl solution was used. The working electrode was coated onto Toray carbon paper with a geometric surface area of approximately 1 cm^2^. The loading of active materials on the working electrode was ∼1.4 mg cm^–2^. Three mL of MgSO_4_ electrolyte (0.1 M) was added to the cell. The chronopotentiometry (CP) galvanostatic charge–discharge measurements were conducted over a voltage range of −0.8 to 0.6 V (vs Ag/AgCl) at a current density of 0.05 A g^–1^. Cyclic voltammetry (CV) measurements were conducted in the homemade cells at scan rates of 0.3 mV s^–1^ between −0.8 and 0.6 V (vs Ag/AgCl). Galvanostatic intermittent titration technique (GITT) measurements were conducted at a current density of 0.025 A g^–1^ for three discharge and charge segments between −0.8 and 0.6 V (vs Ag/AgCl). The discharge and charge times for each step are 10 min, followed by a 10 min relaxation period to reach equilibrium.

### Electron Microscopy

High-Resolution Transmission Electron Microscopy (TEM) was conducted at the Center for Functional Nanomaterials in Brookhaven National Laboratory. The high-angle annular dark field (HAADF) image was acquired using the Thermo Fisher Talos 200× scanning/transmission electron microscope, equipped with an X-FEG electron source module, and operated at 200 keV.

### In Situ X-ray Diffraction (XRD)

The homemade cell was made with an acrylic block and an acrylic cover plate with an O-ring between them for sealing. Cellulose-based filter paper was placed on top of the working electrode, and carbon paper was placed on the back of the filter paper as the support. Such a sandwiched working electrode was placed between the block and the cover plate, where slits of 8 mm × 3 mm were cut for the X-ray beam to pass through. The reference electrode, counter electrode, and exhaust outline channels were built through the top of the acrylic block. The synchrotron XRD studies were conducted at beamline 28-ID-1 at the National Synchrotron Light Source II at the Brookhaven National Laboratory (BNL-NSLS-II). The XRD images were collected on a 2D array detector. In situ XRD measurements were performed in a homemade three-electrode cell as described above with 2 mg loading of Li–V_3_O_8_ ink (1.4 mg Li–V_3_O_8_ active material and 0.6 mg carbon black on 1 cm^2^ carbon paper) as the working electrode, Ag/AgCl as the reference electrode, and Pt wire as the counter electrode. The CV measurement was performed within the potential window from −0.8 V to 0.6 V at a scan rate of 0.3 mV s^–1^ in 0.1 M MgSO_4_ electrolyte. All XRD patterns were subtracted from the background acquired from the bare carbon paper coated with carbon black in the electrolyte. The Rietveld refinement and phase analysis were performed using GSAS-II software. The synchrotron instrument parameters were calibrated by the peak fitting of the CeO_2_ standard. The radiation wavelength is 0.1665 Å.

### X-ray Absorption Spectroscopy (XAS)

XAS measurements were done at beamline 6-BM at the BNL-NSLS-II. To prepare the ex situ XAS samples, the Li–V_3_O_8_ working electrode was first oxidized from open-circuit potential (∼around 0.47 V) to 0.6 V with a scan rate of 0.3 mV s^–1^ in 0.1 M Li_2_SO_4_ electrolyte, then reduced to two reduction states at −0.2 V, −0.8 V, and two oxidation states at 0.2 V, and 0.6 V, respectively. The XAS measurements were carried out in transmission mode at the vanadium K-edge. Vanadium foil and vanadium oxide powders were used as references for X-ray energy calibration and data alignment. Athena software from the Demeter package was used to process and analyze XAS data.

### Debye Scattering Simulation

The simulation of the Debye Scattering intensity for the Li–V_3_O_8_ model was performed using the Easy Pair Distribution Function Fit (EZPIT) Python code kit.

The EZPIT software and instructions are available from https://github.com/NSLS-II-PDF/ezpit?tab=readme-ov-file. The simulated Li–V_3_O_8_ model (3*a* × 9*b* × 4*c*) was built from the NaV_3_O_8_ unit cell (PDF no. 00-016-0601) with five stacking layers along the *a*–*b* plane.

## Results and Discussions

### Electrochemical Characterizations

Figure 1 shows the structural characterization of Li–V_3_O_8_ electrode materials, synthesized by the solution-phase dissolution–crystallization method using commercially available α-V_2_O_5_ bulk materials as the precursor (see synthesis details in the Materials and Methods). Figure 1a exhibits the XRD pattern of Li–V_3_O_8_, showing a monoclinic structure (PDF no. 00-016-0601) with a large interlayer distance (*d*_001_ = 11.3 Å) due to the hydrated Li-ion and water molecules intercalated in the interlayer regions. Broadened diffraction peaks indicate low crystallinity in Li–V_3_O_8_, consistent with previous reports.^11^ Figure 1b depicts the atomic structure of Li–V_3_O_8_, where the Li–V_3_O_8_ molecular layer comprises [VO_6_] octahedra (in blue) and [VO_5_] trigonal pyramids (in green and brown) and is held together by hydrated intercalated Li-ions (in green). Figure 1c shows a high-resolution TEM image of as-made Li–V_3_O_8_, which is crystalline but lacks well-defined long-range order owing to disrupted growth of the layered framework during the solution-phase dissolution–crystallization process.

**Figure 1:**
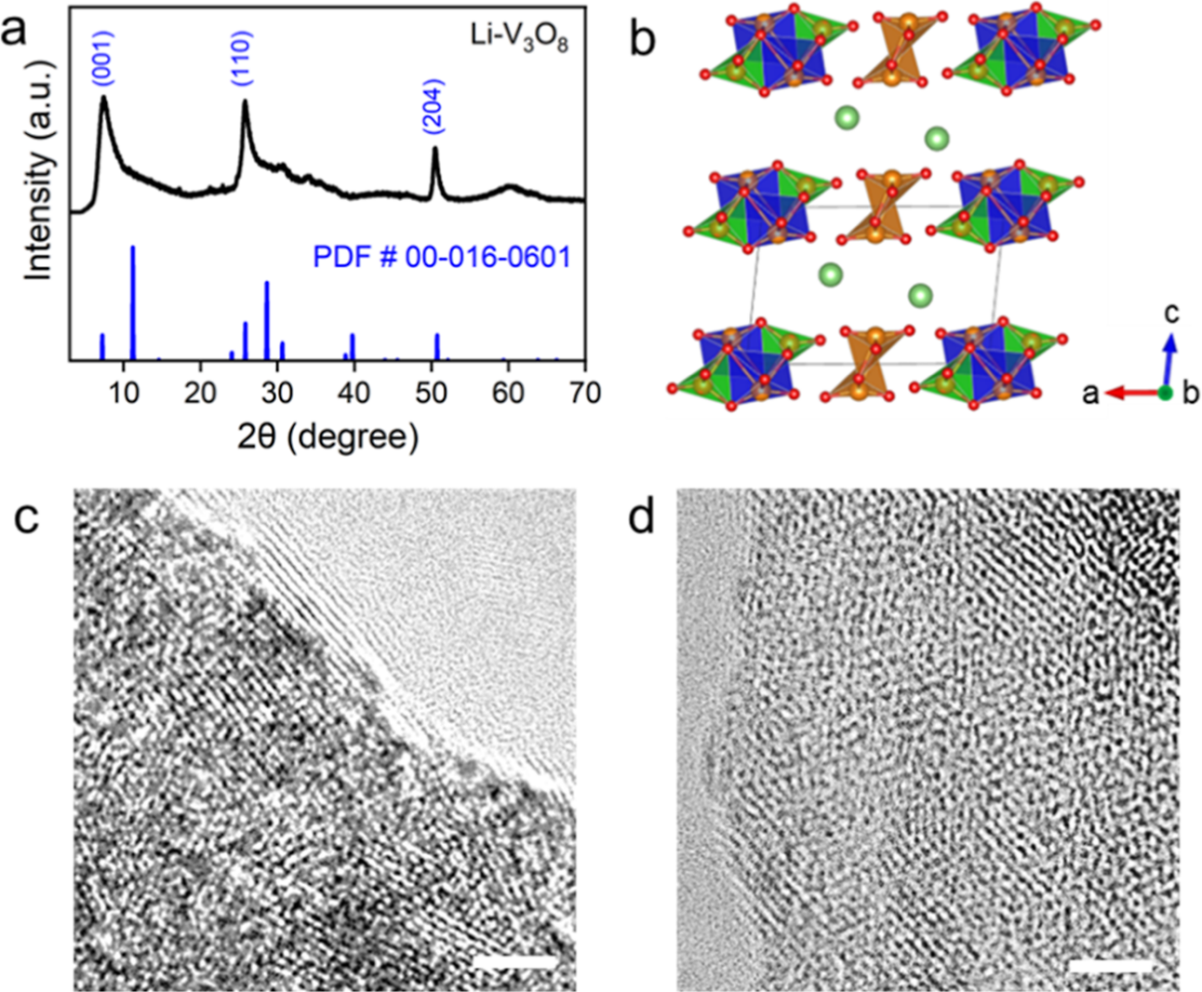
(a) XRD and (b) the atomic structure of Li–V_3_O_8_ materials. TEM images of Li–V_3_O_8_ at (c) pristine state and (d) at the fully discharged state (−0.8 V). Scale bars in (c,d) are 2 nm.

Figure 2 shows the electrochemical properties of Li–V_3_O_8_, examined by CV and CP in 0.1 M MgSO_4_ solution in a homemade three-electrode half-cell with a milligram level of active material loading on the working electrode. Figure 2a shows the CV of the Li–V_3_O_8_ at a scan rate of 0.3 mV s^–1^ with one major reduction peak at −0.34 V (vs Ag/AgCl) in the discharging (reduction) process and one major oxidation peak at −0.18 V in the charging process, suggesting a single-stage Mg^2+^ intercalation and deintercalation. Notably, Li–V_3_O_8_ exhibits broadened redox peaks upon Mg^2+^ intercalation and deintercalation, consistent with its highly disordered crystalline structure, as observed from HR-TEM and XRD. Figure 2b shows the CP measurement conducted at a current density of 0.05 A g^–1^ between −0.8 and 0.6 V, which reveals the first-cycle reduction (Mg^2+^ insertion) capacity of 140 mAh g^–1^, corresponding to 0.17 electron transfer per vanadium atom. An additional oxidation peak is also discernible at ∼0.1 V from CV (Figure 2a), CP (Figure 2b), and first order derivative of charge over potential (dQ/dV) plot (Figure 2b), which shows the gradual disappearance of this peak over extended cycling (Figure 2c). While the exact origin of this peak is not fully understood, it might suggest Li^+^ transport within the Li–V_3_O_8_ host (intercalation or deintercalation) is affected by various energy barriers strongly associated with the local coordination environment of the V–O layers. The gradual disappearance of this peak over cycling suggests a transient structural rearrangement or metastable transport pathway that diminishes as the electrode structure stabilizes during repeated charge–discharge cycles.

**Figure 2:**
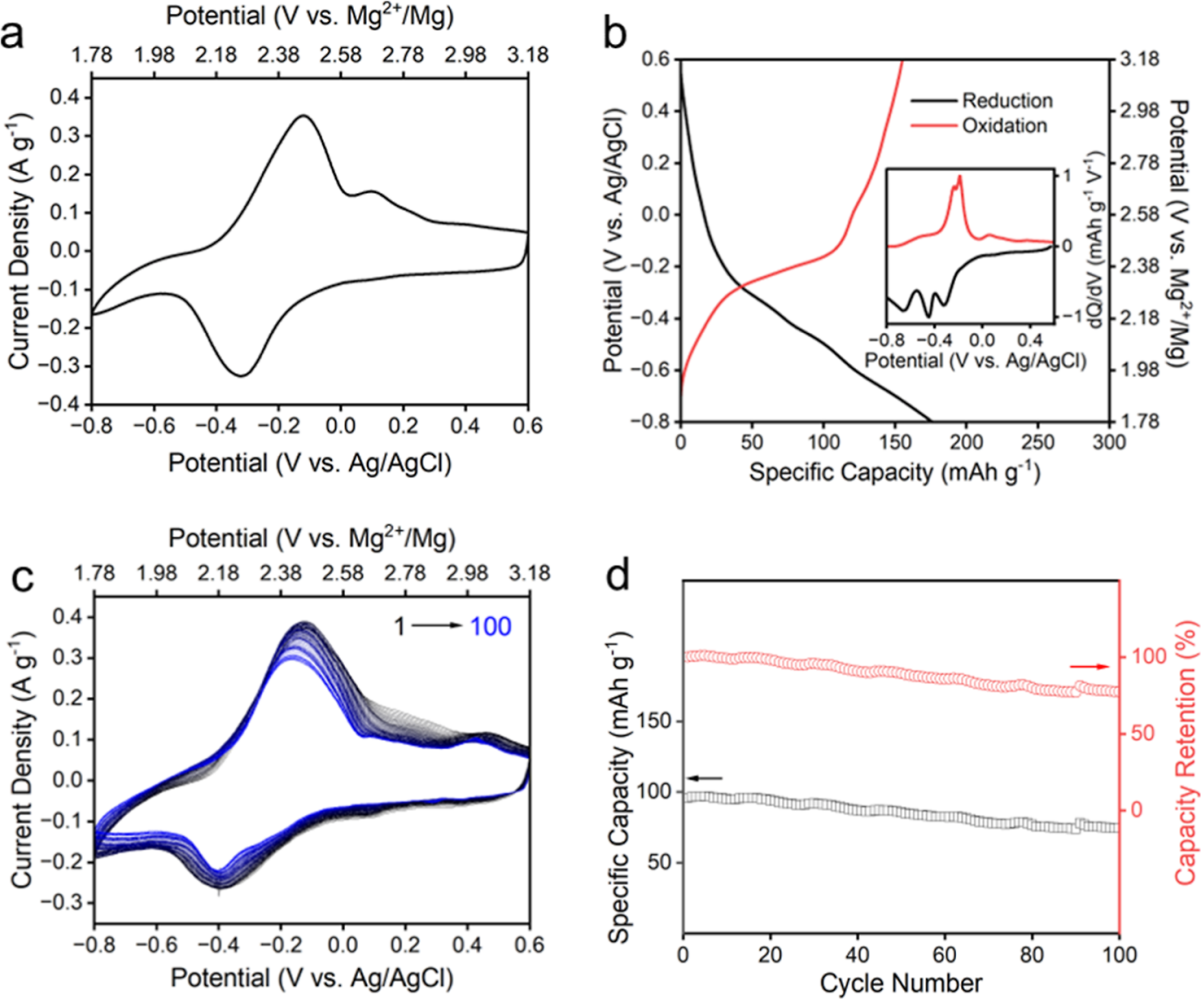
(a) CV and (b) CP plots of Li–V_3_O_8_ in 0.1 M MgSO_4_ solution. The inset shows 1st order derivative of charge over potential (dQ/dV) plot. (c) CV plots over 100 cycles in vanadium-ion-balanced 0.1 M MgSO_4_ solution, and (d) corresponding reduction capacity and capacity retention. CV was conducted at a scan rate of 0.3 mV/s in (a) and 0.5 mV/s in (c), and CP is at a current density of 0.05 A/g.

Figure S1 shows the high-angle annular dark-field (HAADF) image and the corresponding Mg and V elemental maps of the Li–V_3_O_8_ electrode obtained at −0.8 V in 0.1 M MgSO_4_ solution, acquired using scanning transmission electron microscopy (STEM) equipped with energy-dispersive X-ray spectroscopy (EDS). Two representative regions are presented for imaging and elemental mapping. Both sets of results confirm the layered morphology of the Li–V_3_O_8_ electrode and the homogeneous distribution of Mg throughout the Li–V_3_O_8_ structure.

In addition to Mg^2+^ storage, we also conducted electrochemical measurements on Li–V_3_O_8_ in 0.1 M Li_2_SO_4_ and Na_2_SO_4_ electrolytes for comparison (Figure S2). The results show that Li–V_3_O_8_ exhibits the highest Mg^2+^ storage capacity in the first cycle of the CP measurement (140 mAh g^–1^), compared with Na^+^ (114 mAh g^–1^) and Li^+^ (94 mAh g^–1^) under the same voltage window. Further research is needed to elucidate the intercalation chemistry of alkali and multivalent ions in Li–V_3_O_8_, where hydration energy, ionic radius, and the interlayer spacing of the Li–V_3_O_8_ structure might all play a role.

Notably, dissolution has been the primary cause of poor capacity retention in vanadium oxide electrodes in aqueous electrochemical systems, owing to its higher water solubility relative to other transition-metal oxide and hydroxide materials. In this study, we adopt a common-ion suppression approach, following the Le Châtelier principle, to mitigate Li–V_3_O_8_ dissolution by adding 7 mM vanadium ions (equivalent to the vanadium ion concentration at saturation in water at room temperature). If Li–V_3_O_8_ dissolves, a deliberate vanadium-ion reservoir allows the reverse process (redeposition) onto the electrode. This helps maintain mass balance and slows capacity fade. A similar approach has been used for other aqueous electrode systems that also suffer from electrode dissolution, especially manganese- and vanadium-based electrodes.^30–32^ Figure 2c shows 100 cycles of CV measurements conducted at a current density of 0.5 mV s^–1^ in vanadium-ion-balanced 0.1 M MgSO_4_ electrolyte, showing an average discharge capacity retention of 75.2%, comparable to or superior to the reported capacity retention of divalent ions (e.g., Mg^2+^, Zn^2+^) in aqueous systems.^33^ In comparison, Li–V_3_O_8_ exhibits severe capacity loss (∼72% retention after 25 CV cycles) without vanadium-ion additives (Figure S3). CV was conducted to evaluate the effects of the dissolved vanadium ions in the balanced electrolyte on overall charge storage in Li–V_3_O_8_ electrode materials. Figure S4 shows CV curves in the vanadium-ion-balanced electrolyte, with no discernible redox features attributable to vanadium ion insertion/extraction within the tested potential window. The observed featureless current response is consistent with capacitive interactions between the electrolyte and electrode surface. These results suggest that the charge storage process is dominated by Mg^2+^ insertion/extraction in the electrode material rather than Faradaic reactions involving dissolved vanadium ions.

### In Situ XRD Reveals Intercalation-Induced Amorphization of Vanadate Electrode

In situ synchrotron XRD is used to examine the structural evolution of Li–V_3_O_8_ during Mg^2+^ intercalation and deintercalation. Figure 3a shows the CV measurements between −0.8 and 0.6 V at a scan rate of 0.3 mV s^–1^, where diffraction patterns of Li–V_3_O_8_ were collected simultaneously. The waterfall and contour diffraction patterns shown in Figure 3b,c suggested no new phase generation when Li–V_3_O_8_ was cycled, confirming the formation of solid-solution-type Mg^2+^ intercalated vanadate compounds. Figure 3d shows the zoomed-in regions of the contour plots of the (001), (110), and (204) diffraction peaks during the CV cycling. As shown in Figure 3d,e, when the Li–V_3_O_8_ is reduced from 0.6 V to −0.8 V, the (204) peak shifted to lower 2θ angles, suggesting the increased interplanar spacing of the (204) plane (*d*_204_). During sequential charging from −0.8 to 0.6 V, the (204) peak shifted to higher 2θ angles and showed decreased *d*_204_ values. The reversible changes in the diffraction peaks suggested the expansion of the Li–V_3_O_8_ lattice upon Mg^2+^ intercalation (reduction), because reduced vanadium ions have an increased ionic radius and V–O bond length, and thus a larger interplanar distance. Accordingly, when the potential increased from −0.8 to 0.6 V (oxidation), the Li–V_3_O_8_ lattice contracted during Mg^2+^ deintercalation. Similar trends are observed in the evolution of (110) peaks, where reversible lattice expansion and contraction occur upon the Mg^2+^ intercalation and deintercalation, respectively, owing to the reduction and oxidation of vanadium ions. On the other hand, the basal diffraction peak, such as (001), shows trends opposite to those of the (204) and (110) Bragg diffraction peaks, where Mg^2+^ intercalation within the interlayer spacing strengthen the electrostatic interaction between the positively charged Mg^2+^ ions and negatively charged V–O molecular layers, showing narrowed interlayer spacing, and thus basal diffraction peak shifted to a higher 2θ angle, and vice vera during the Mg^2+^ deintercalation.

**Figure 3:**
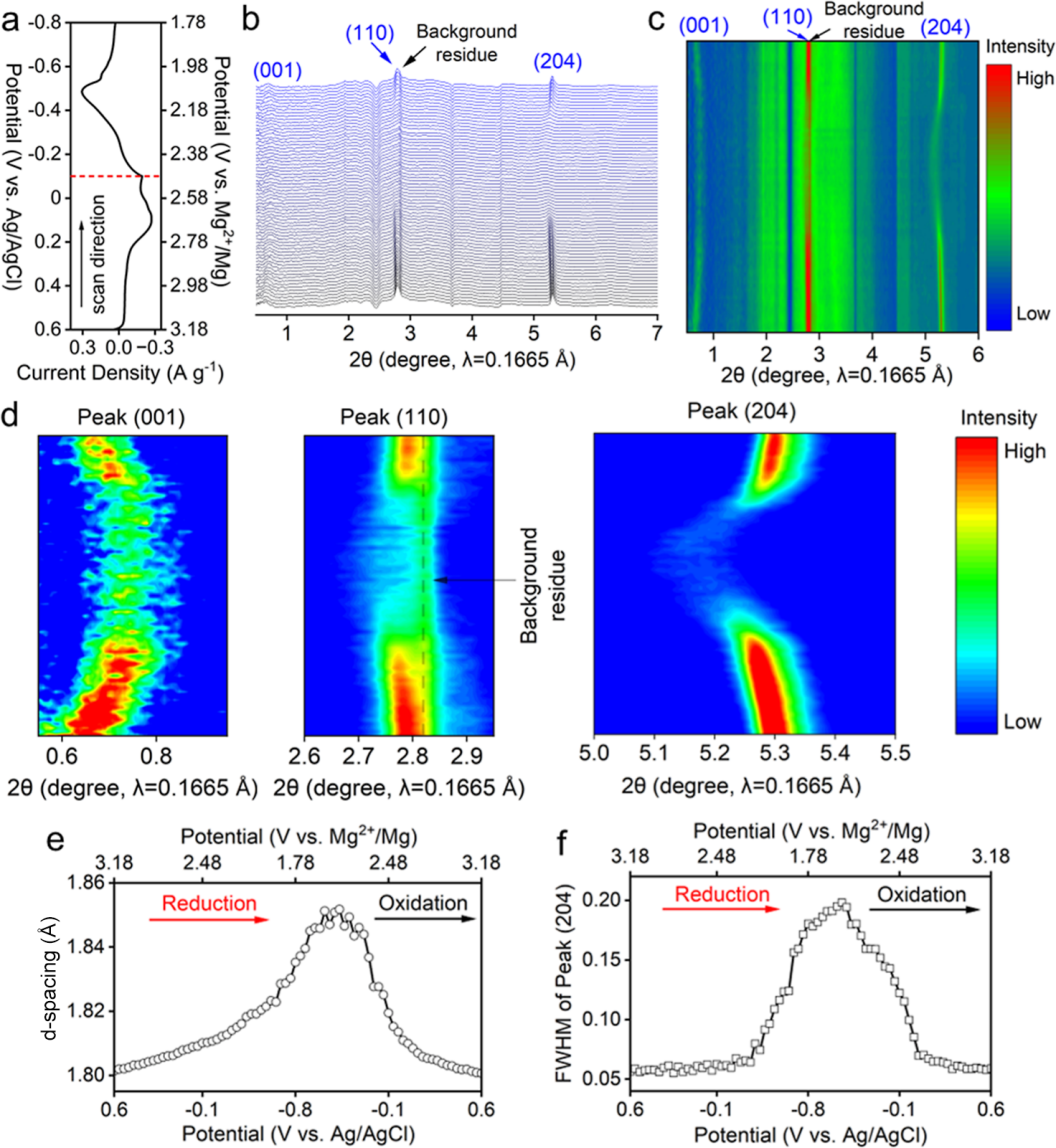
(a) CV for in situ XRD, (b) waterfall and (c) contour plots of in situ spectra, (d) zoomed-in regions of peaks (001), (110), and (204), (e) *d*-spacing and (f) fwhm evolutions of peak (204).

More interestingly, in situ XRD reveals the reversible amorphization-crystallization of Li–V_3_O_8_ induced by Mg^2+^ intercalation and deintercalation. Figure 3f shows that during the initial Mg^2+^ intercalation from 0.6 V to −0.8 V, the full width at half-maximum (fwhm) of the (204) peak is around 0.05°. The constant fwhm suggests negligible Mg^2+^ intercalation. When the potential decreased from −0.2 V to −0.8 V, the fwhm of (204) increased rapidly from 0.05° to 0.20°, accompanied by a nearly vanishing (204) and (110) peaks. This suggests that Mg^2+^ intercalation induces turbostratic disordering and amorphization in Li–V_3_O_8_ materials. When Mg^2+^ intercalates into the van der Waals gap between V–O molecular frameworks, the increasing electrostatic interaction between Mg^2+^ and the negatively charged V–O layers causes the random stacking of V–O layers. Sequentially, a distinctive amorphization-to-crystallization structural restoration was observed when the Li–V_3_O_8_ was continuously oxidized from −0.8 V to 0.6 V, during which the (204) peak reappeared and intensified rapidly. This suggests a structural ordering of the Li–V_3_O_8_ electrode during Mg^2+^ deintercalation, weakening the electrostatic interaction between adjacent V–O layers and relieving turbostratic disorder. It is important to point out that amorphization in materials can arise from multiple structural origins. In the present case, we consider the observed amorphization primarily reflects the loss of long-range stacking order (turbostratic disorder) rather than complete atomic-scale amorphization (e.g., loss of both long-range periodicity and local structural order), as elucidated by the Debye scattering simulation in the later discussion.

### Kinetics Analysis Suggests Electrode Amorphization Promotes Mg^2+^ Transport

To further investigate the kinetics of Mg^2+^ intercalation, we performed galvanostatic intermittent titration technique (GITT) on Li–V_3_O_8_ as shown in Figure 4. The diffusion coefficient (*D*) can be calculated according to eq 1

1 $$D=\frac{4}{\pi \tau }{\left(\frac{n_{m}V_{m}}{S}\right)}^{2}{\left(\frac{\Delta E_{s}}{\Delta E_{t}}\right)}^{2}$$

where τ, mB, VM, MB, and *S* are the constant current pulse time, mass, molar volume, molar mass, and electrode–electrolyte interface area of Li–V_3_O_8_, respectively. Δ*E*_s_ is the voltage difference during the open circuit period, and Δ*E*_t_ represents the total change of cell voltage during a constant current pulse, excluding the IR drop.

**Figure 4:**
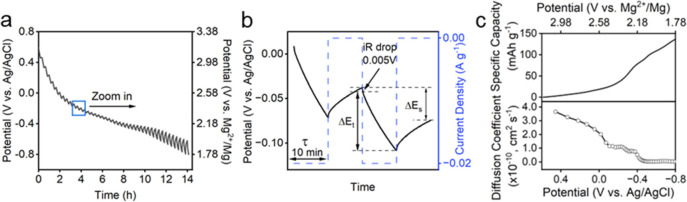
(a) GITT plot of Li–V_3_O_8_ during discharge process, (b) zoom-in area to show parameters in eq 1, (c) calculated diffusion coefficient (bottom) with corresponding discharge process (top).

The calculated result shows that Mg^2+^ diffusivity rapidly decreases from ∼2 × 10^–10^ to ∼5 × 10^–11^ cm^2^ s^–1^ from 0.6 to 0 V at the early stage of Li–V_3_O_8_ reduction, during which only minor Mg^2+^ intercalation occurs. The decrease in the diffusion coefficients is likely due to the formation of energy barriers arising from structural reorganization during the order-to-disorder transition upon Mg^2+^ intercalation. As the potential decreases from 0 V to −0.8 V, significant Mg^2+^ intercalation occurs, as evidenced by the CV and in situ XRD analyses. The diffusion coefficients decrease because of the charge repulsion from the increase in Mg^2+^ concentration in the Li–V_3_O_8_ host. However, as the potential decreases to 0 V, the decrease in diffusion coefficients slows. Notably, in situ XRD and fwhm analyses indicate that at a potential of −0.4 V, Mg^2+^-intercalated Li–V_3_O_8_ becomes nearly amorphous. Interesting, the GITT analysis shows that when Li–V_3_O_8_ experienced severe amorphization and the Mg^2+^ diffusion coefficient becomes distinctly stagnant, and remains unchanged still the −0.8 V. Notably, during this nearly amorphous state, nearly 3/7 of total Mg^2+^ ions intercalated into Li–V_3_O_8_ (corresponding to ∼60 mAh/g Mg^2+^ storage capacity between −0.4 V and −0.8 V), strongly suggesting the distortive local structure facilitates Mg^2+^ diffusion into the vanadate electrode materials, thereby improving storage capacity. Collectively, GITT, in situ XRD, and electrochemical analyses provided explicit evidence for the connection between turbostratic disorder/amorphization and improved Mg^2+^ intercalation capability.

Notably, the order of magnitude of the Mg^2+^ diffusion coefficients obtained in this work is consistent with the values reported in our previous study on Zn^2+^ transport in Li–V_3_O_8_,^11^ where similarly slow ion diffusion was observed within the layered framework. It is important to point out that Mg^2+^ has a very high hydration energy compared with many other cations. The strong Mg^2+^–water interaction may influence diffusion kinetics and contribute to the relatively low diffusion coefficients observed.

Post-mortem TEM analysis of fully discharged Li–V_3_O_8_ also supports the intercalation-induced amorphization process, as shown in Figure 1d. Compared with the as-made Li–V_3_O_8_, the intercalated electrode exhibited misaligned lattice fringes that were still discernible in certain regions but appeared discontinuous and irregular, indicating a highly disordered or nearly amorphous structure. This distorted structure facilitated ion transport by increasing interlayer spacing and generating abundant diffusion pathways within the material, supported by in situ XRD and GITT analyses.

### Debye Scattering Simulation to Understand Intercalation-Induced Amorphization

Li–V_3_O_8_ materials comprise partially disconnected [VO_5_] square pyramids and [VO_6_] octahedra, allowing local relaxation of layer distortions. Such structural flexibility facilitates turbostratic displacements to facilitate Mg^2+^ transport, as suggested in the above discussion. To obtain an understanding of turbostratic disorder and amorphization of Li–V_3_O_8_ materials induced by Mg^2+^ intercalation on an atomic level, we employed the Debye scattering simulation to evaluate various displacements of V–O layers (e.g., random rotations, translational shifts, oscillatory motions, or varied interlayer spacing). The scattering intensity is calculated by summing the contributions of individual atoms and their interatomic distances to quantify the influence of positional perturbations of V and O atoms, resulting from turbostratic distortions/amorphization, on the scattering intensity.

We first constructed various Li–V_3_O_8_ models by stacking planar V–O layers with lateral dimensions ranging from 1.2 to 3.7 nm and thicknesses ranging from 1.6 to 4.0 nm. Figure S5 shows that as the lateral dimensions and the number of stacked layers increased, the simulated diffraction patterns exhibited more prominent peaks, as expected. Considering the balance between computational cost and simulation reliability. We selected a cell dimension of 3.7 nm × 3.2 nm × 3.2 nm, corresponding to a supercell containing 3240 atoms and five stacked V–O layers. Turbostratic configurations were generated from this 5-layer model by manipulating the second and fourth V–O layers to introduce controlled translation (Figure 5a), rotation (Figure 5b), oscillation (Figure 5c), and interlayer spacing variations (Figure 5d), and the calculated scattering intensities of the (204) peak at the extent of displacement are exhibited in Figure 5e–h, respectively. Specifically, layer translation was simulated by sliding the two layers along the *a*-axis in opposite directions; layer rotation was implemented by rotating the layers in opposite directions about the *c*-axis; layer oscillation was simulated by opposite-direction rotations about the *b*-axis; and interlayer distance variation was introduced by adjusting the separation along the *c*-axis. The simulated scattering curves (Figures 5e–h) demonstrate that each type of displacement is reflected in the diffraction profile, with maximum reductions of 28%, 15%, 22%, and 22% for layer translation, rotation, oscillation, and varied interlayer spacing, respectively. Notably, these individual displacement modes do not offer the experimentally observed intensity attenuation and fwhm widening when acting alone. Therefore, the experimentally observed amorphization during Mg^2+^ intercalation likely arises from a more complex combination of multiple displacement modes, driven by strong electrostatic interactions between the divalent Mg^2+^ ions and the negatively charged host layers.

**Figure 5:**
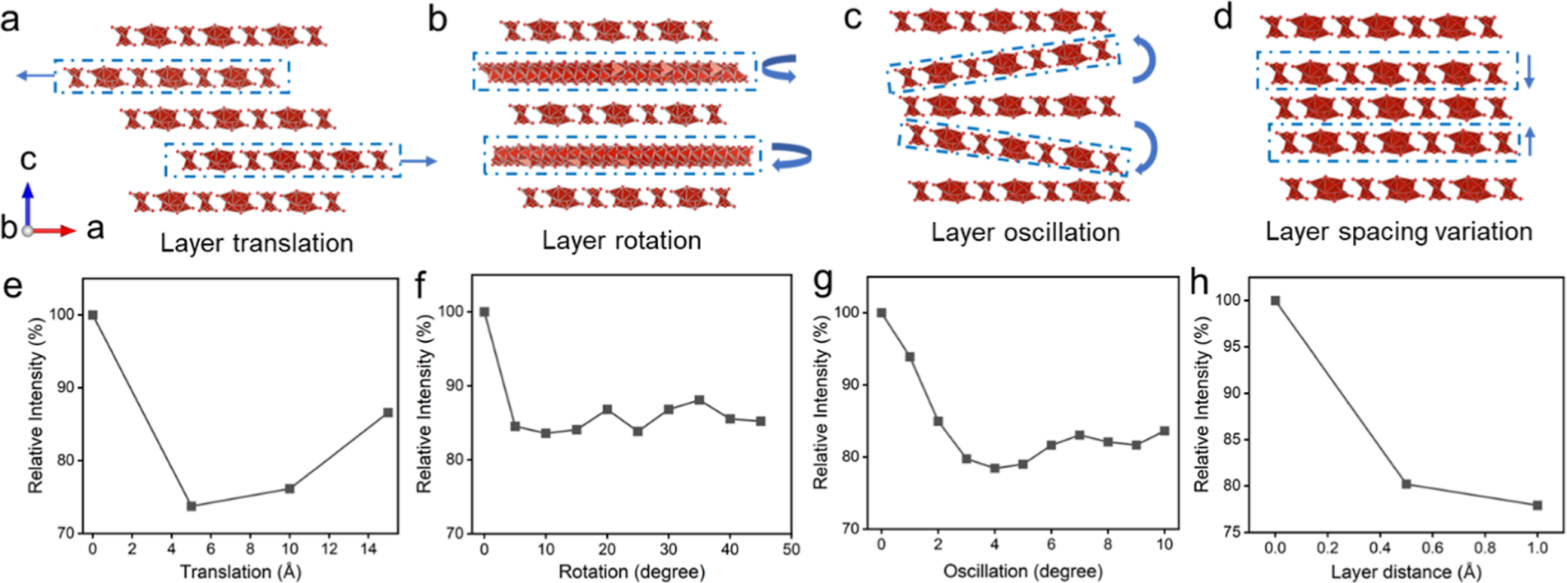
Layer displacement illustration of (a) translation, rotation, oscillation, and spacing variation, and (b) their effects on the relative intensity changes of Peak (204).

### Ex Situ XAS

To elucidate the electronic changes accompanying Mg intercalation, we monitored the evolution of the vanadium oxidation state using X-ray absorption near-edge spectroscopy (XANES) at several electrochemical states: open-circuit potential (OCP, initial state), −0.2 V (partially reduced), −0.8 V (fully reduced), 0.2 V (partially oxidized), and 0.6 V (fully oxidized), as shown in Figure 6. These spectra were compared with V_2_O_4_ and V_2_O_5_ standards, which provide well-defined vanadium valence states. During Mg intercalation, the vanadium K-edge absorption energy at the half-edge step [1/2 μ­(E)] systematically shifted to lower energies, reaching a minimum at the most reduced state. This evolution indicates a progressive reduction of vanadium. Upon Mg deintercalation, the 1/2 μ­(E) position shifted back toward higher energies, consistent with reoxidation of vanadium. Changes in the pre-edge feature provide additional insight into the local V–O coordination environment. In particular, the pre-edge region near 5470.4 eV is highly sensitive to distortions in the [VO_6_] octahedral geometry. The Li–V_3_O_8_ structure consists of both asymmetric [VO_5_] square pyramids and symmetric [VO_6_] octahedra; the former exhibits greater distortion and therefore produces stronger pre-edge intensities. Upon reduction, electrons populate the hybridized V 3d–O 2p states, converting V^5+^ to V^4+^ and promoting the structural transformation of distorted [VO_5_] into more symmetric [VO_6_]. Correspondingly, along with vanadium reduction, the pre-edge intensity decreased, the fwhm broadened, and the pre-edge peak position shifted to lower energy. During subsequent oxidation, these features are restored to their initial features, reflecting the reformation of distorted [VO_5_] from the more symmetric [VO_6_]. Taken together, the evolution of both the pre-edge structure and the 1/2 μ­(E) energy shift confirms that a reversible V^5+^/V^4+^ redox couple operates during Mg^2+^ intercalation and deintercalation in Li–V_3_O_8_.

**Figure 6:**
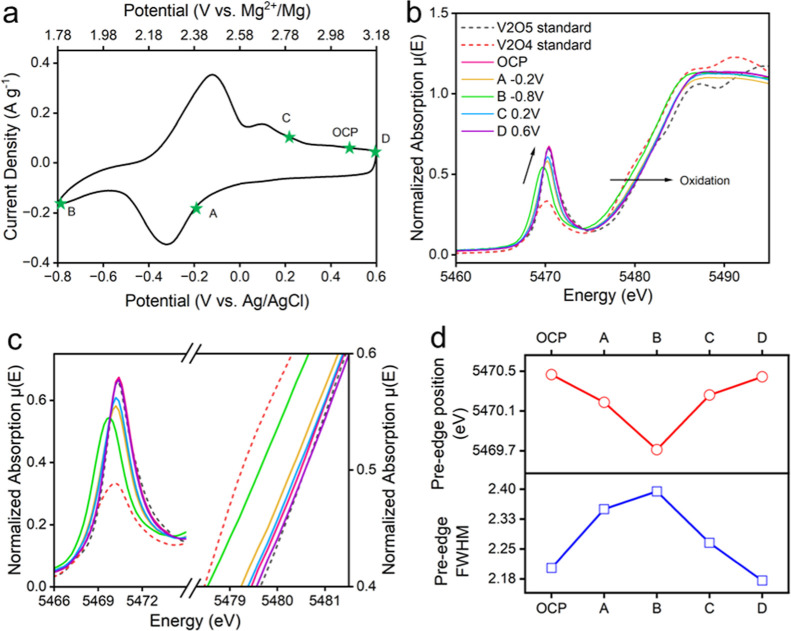
(a) The chosen electrochemical states that are used for ex situ XAS measurements, (b) XANES of Li–V3O8 at different potentials from (a), (c) zoomed-in regions to present pre-edge and white line feature from (b), and (d) the pre-edge positions and their fwhm values at different chosen points.

## Conclusions

We have reported a reversible amorphization-crystallization transition of Li–V_3_O_8_ electrode materials induced by Mg^2+^ intercalation and deintercalation in an aqueous electrolyte. Detailed characterization, including in situ XRD, XAS, TEM, electro-kinetics, and Debye scattering simulation, has shown that intercalation-induced turbostratic disorder, especially random rotations, translational shifts, oscillatory motions, or varied interlayer spacing of adjacent V–O molecular layers, could be responsible for the observed amorphization. More importantly, various characterization tools collectively demonstrated that the distorted local structure facilitated Mg^2+^ intercalation, with nearly 3/7 of the total intercalated Mg^2+^ attributable to structural amorphization. Several layered battery materials have been reported to exhibit intercalation-induced turbostratic disorder and amorphization.^12^ However, few have observed a reversible amorphization-crystallization transition in aqueous Mg^2+^-hosting electrodes and explicit structural and electrochemical evidence to connect the structural amorphization and improved ion storage capability, as shown in this work. The study presented reveals an intriguing relationship between ion transport and the reversible amorphization-to-crystallization dynamics it induces, opening a paradigm for designing advanced aqueous battery electrodes.

## Supplementary Material



## Notes

HAADF images along with the elemental mapping of V and Mg, additional electrochemical characterizations, and the different Li–V_3_O_8_ crystalline models along with their simulated scattering patterns (PDF)
