# Supplementary material for: Intercalation-Induced Amorphization Boosts Aqueous Magnesium-Ion Storage
Source: Chem Mater. 2026 Apr 21;38(9):4570–9. doi: 10.1021/acs.chemmater.5c03477 (PMC13173499; doi:10.1021/acs.chemmater.5c03477)
Supplement: Supplementary file 1 [file cm5c03477_si_001.pdf]

Supporting Information for

Intercalation-Induced Amorphization Boosts Aqueous Magnesium-Ion Storage

Tongxin Zhou,[a] Divakar Arumugam,[a] AM Milinda Abeykoon,[b] Gihan Kwon,[b] Cheng-Hung Lin,[b] Lihua Zhang,[c] and Xiaowei Teng[a]*

a Department of Chemical Engineering, Worcester Polytechnic Institute, 100 Institute Road, Worcester, MA 01609, United States

b National Synchrotron Light Source II, Brookhaven National Laboratory, Upton, New York 11973, United States.

c Center for Functional Nanomaterials, Brookhaven National Laboratory, Upton, New York 11973, United States

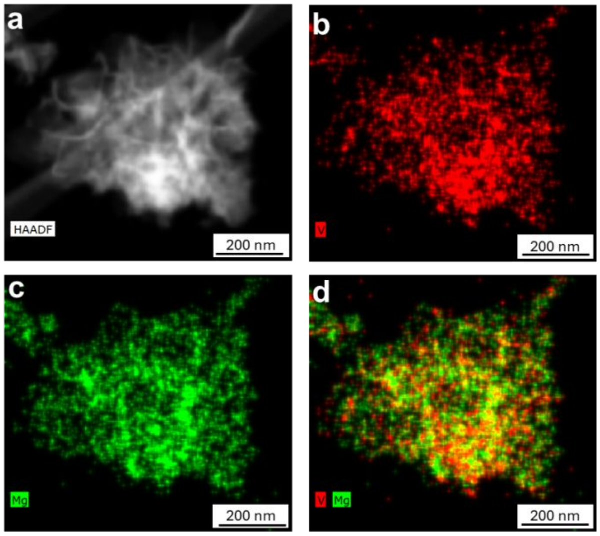

Figure S1a. HAADF image and the elemental mapping from two representative regions. (a) HAADF image, EDS mapping of (b) V (red), (c) Mg (green), and (d) combined Mg and V in the same image.

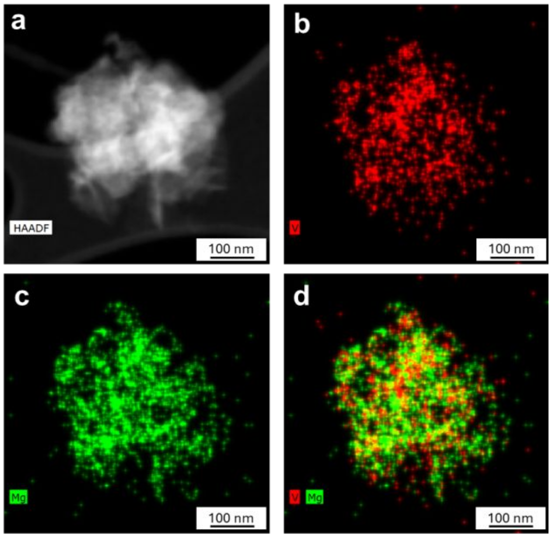

Figure S1b. HAADF image and the elemental mapping from two representative regions. (a) HAADF image, EDS mapping of (b) V (red), (c) Mg (green), and (d) combined Mg and V in the same image.

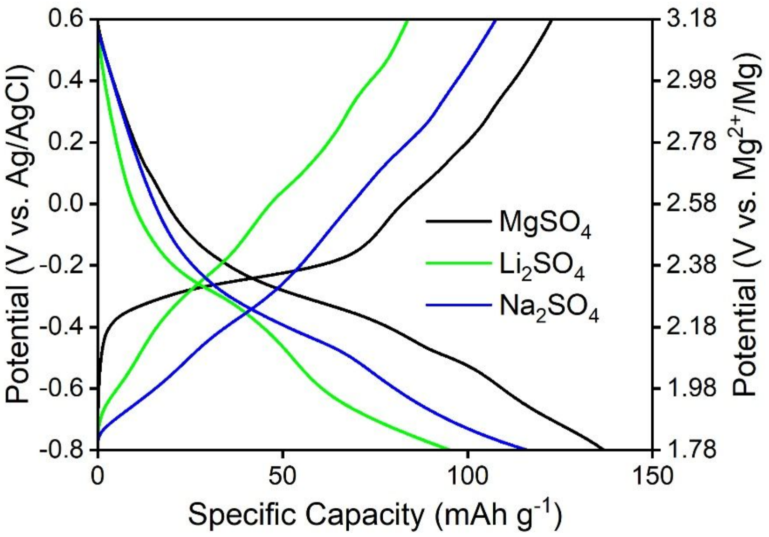

Figure S2. CP measurements of Li-V_3O_8 in 0.1 M Li_2SO_4 and Na_2SO_4 electrolytes

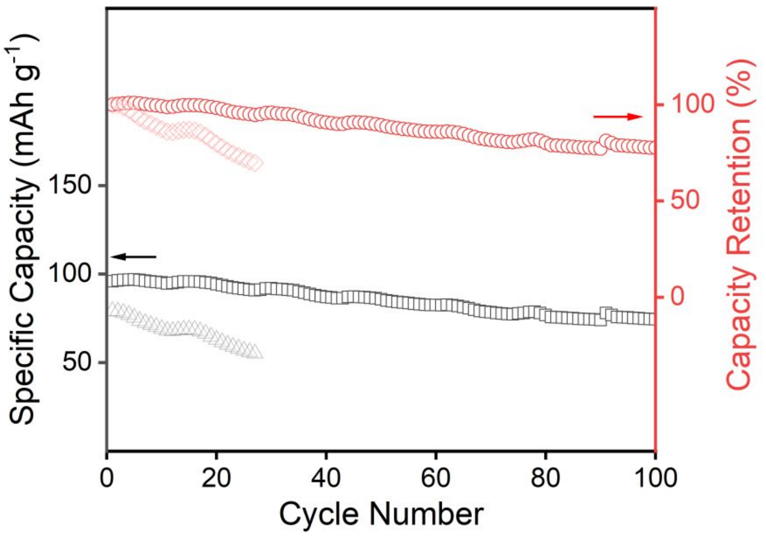

Figure S3. Capacity fades across cycles compared between 0.1M $MgSO_4$ electrolytes with (dark dots) and without (light dots) vanadium-ion additives.

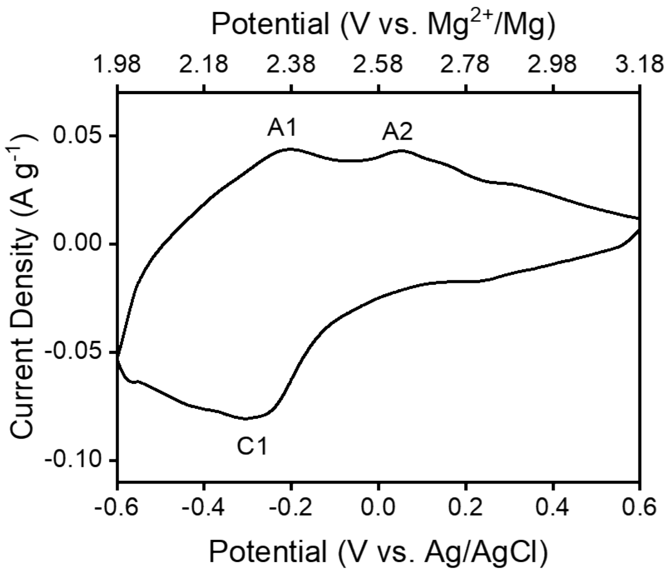

Figure S4. CV curves in the vanadium-ion-balanced electrolyte.

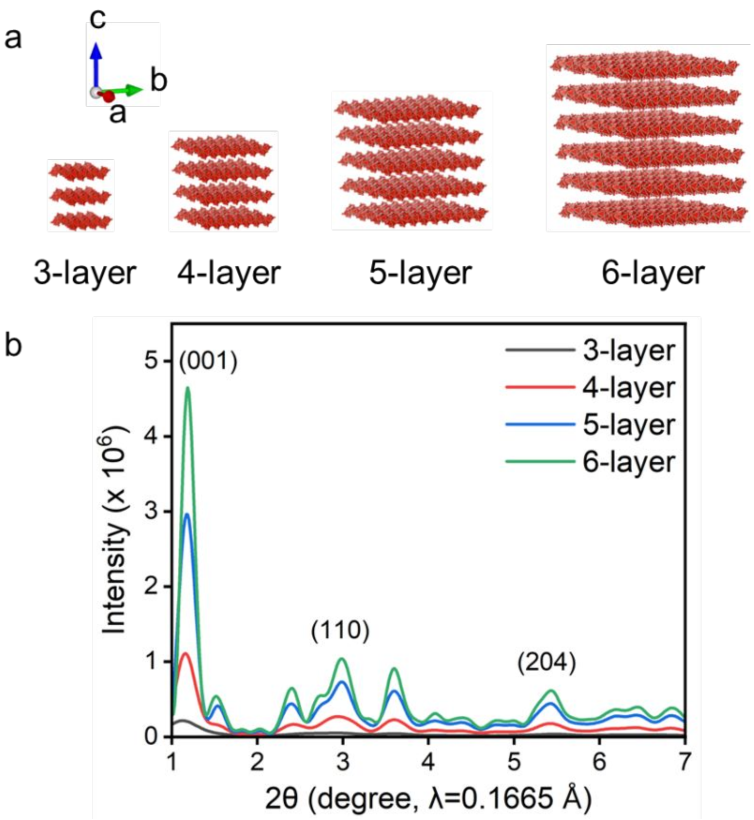

Figure S5. Ideal Li-V_3O_8 crystalline structure build-up with (a) different layers, and (b) the corresponding scattering patterns calculated from the Debye equation.
